# Wafer-Level Fabrication of Radiofrequency Devices Featuring 2D Materials Integration

**DOI:** 10.3390/nano15141119

**Published:** 2025-07-18

**Authors:** Vitor Silva, Ivo Colmiais, Hugo Dinis, Jérôme Borme, Pedro Alpuim, Paulo M. Mendes

**Affiliations:** 1 CMEMS—Center for Microelectromechanical Systems, University of Minho, Campus de Azurém, 4800-058 Guimarães, Portugalhdinis@dei.uminho.pt (H.D.); 2 LABBELS—Associate Laboratory, 4710-057 Braga, Portugal; 3 INL—International Iberian Nanotechnology Laboratory, Av. Mestre José Veiga, 4715-330 Braga, Portugal; jerome.borme@inl.int (J.B.); pedro.alpuim.us@inl.int (P.A.); 4 Center of Physics, University of Minho, Campus de Gualtar, 4710-057 Braga, Portugal

**Keywords:** graphene, radiofrequency, GFET, antennas, nanofabrication

## Abstract

Two-dimensional (2D) materials have been proposed for use in a multitude of applications, with graphene being one of the most well-known 2D materials. Despite their potential to contribute to innovative solutions, the fabrication of such devices still faces significant challenges. One of the key challenges is the fabrication at a wafer-level scale, a fundamental step for allowing reliable and reproducible fabrication of a large volume of devices with predictable properties. Overcoming this barrier will allow further integration with sensors and actuators, as well as enabling the fabrication of complex circuits based on 2D materials. This work presents the fabrication steps for a process that allows the on-wafer fabrication of active and passive radiofrequency (RF) devices enabled by graphene. Two fabrication processes are presented. In the first one, graphene is transferred to a back gate surface using critical point drying to prevent cracks in the graphene. In the second process, graphene is transferred to a flat surface planarized by ion milling, with the gate being buried beneath the graphene. The fabrication employs a damascene-like process, ensuring a flat surface that preserves the graphene lattice. RF transistors, passive RF components, and antennas designed for backscatter applications are fabricated and measured, illustrating the versatility and potential of the proposed method for 2D material-based RF devices. The integration of graphene on devices is also demonstrated in an antenna. This aimed to demonstrate that graphene can also be used as a passive device. Through this device, it is possible to measure different backscatter responses according to the applied graphene gating voltage, demonstrating the possibility of wireless sensor development. With the proposed fabrication processes, a flat graphene with good quality is achieved, leading to the fabrication of RF active devices (graphene transistors) with intrinsic f_T_ and f_max_ of 14 GHz and 80 GHz, respectively. Excellent yield and reproducibility are achieved through these methods. Furthermore, since the graphene membranes are grown by Chemical Vapor Deposition (CVD), it is expected that this process can also be applied to other 2D materials.

## 1. Introduction

Graphene has been widely presented as a wonder material with extraordinary properties, with a wide range of applications and the potential to revolutionize various industries. One possible application of graphene is electronic circuits, more specifically for RF applications. Graphene’s properties originate from its unique honeycomb lattice that is formed by a single sheet of ${sp}^{2}$-hybridized carbon atoms, resulting in a zero-bandgap semiconductor. This allows graphene to have a relatively high conductivity, low resistance, sustain a high current density [1], and possess high carrier mobility that can reach up to 200,000 cm^2^ V^−1^s^−1^ [2]. Graphene RF transistors have the potential to achieve cutoff frequencies in the hundreds of GHz [3]. It becomes apparent that, by integrating graphene and exploring its properties, it should be possible to improve electrical circuits [1,4,5].

However, there are various limitations in the fabrication of graphene-based devices. An example of this is transistors, whose performance is typically severely degraded. One of the reasons for this is the fact that high-κ gate dielectrics are required to maximize the RF performance. However, it is difficult to grow a high-κ oxide on top of graphene due to the lack of reactive sites for Atomic Layer Deposition (ALD), especially when thin oxide layers are required. Another reason is the high contact resistance between graphene and its metal contact [6].

To improve RF performance, several techniques can be implemented: the use of physical gates to increase the f_max_ of the transistors [7], or complex gate structures designed to reduce parasitic and access resistances [8]. Self-aligned structures are also being developed to minimize access resistance, thereby improving the RF performance of GFETs [9], which is a critical parameter for achieving high-performance graphene RF oscillators.

A comprehensive review on graphene applications in the design of RF building blocks, their performance, and challenges is presented by the authors in [6]. Additionally, this review approaches RF transistor state of the art, detailing applications such as oscillators, multipliers, and mixers. Finally, the review also discusses fabrication techniques and issues, providing a comprehensive overview of graphene for RF applications.

In this work, the design and fabrication of graphene devices is discussed. Two fabrication approaches are presented: a buried bottom-gate graphene device fabrication process without surface planarization, using a critical point dryer to achieve crack-free graphene transfer, and a surface planarization technique employing ion milling prior to graphene transfer. These techniques have the objective of preserving the graphene lattice so that high carrier mobilities can be achieved. In both cases, Chemical Vapor Deposition (CVD)-grown graphene is used, as well as the PMMA-assisted transfer method. The performance of devices produced with both fabrication approaches is assessed and compared, allowing for a better understanding of the performance differences between the two approaches.

To demonstrate the development of graphene RF devices, the surface planarization technique with ion milling prior to graphene transfer is used to fabricate an inverter and a nonlinear block. Finally, to demonstrate the application of the fabrication process on the development of RF electronics, graphene is included as a passive element on an antenna, allowing for a backscatter readout that can be implemented in electronic sensors.

The manuscript is organized as follows. Section 1 is an introduction. Section 2 describes two graphene RF device fabrication techniques. Section 3 contains the experimental validation of the RF graphene transistors fabricated with both the methods proposed in Section 2. Section 4 describes the development of RF circuits, such as an oscillator, with graphene transistors, as well as the development and testing of passive RF graphene components. Section 5 presents this work’s conclusions.

## 2. Methodology

As previously discussed, two approaches to fabricating active devices (RF graphene transistors) and passive devices such as coils, antennas, and capacitors will be presented in this work. First, we present a bottom-gate graphene device fabrication process without surface planarization, along with a detailed discussion of the associated challenges. To achieve a crack-free graphene transfer, a critical point-drying process was developed and employed to dry the samples containing the devices, preserving the graphene lattice characteristics. In the other approach, a surface planarization technique using ion milling prior to graphene transfer was applied, resulting in the successful fabrication of a buried gate RF graphene FET, demonstrating the effectiveness of the planarization process.

**Figure 1:**
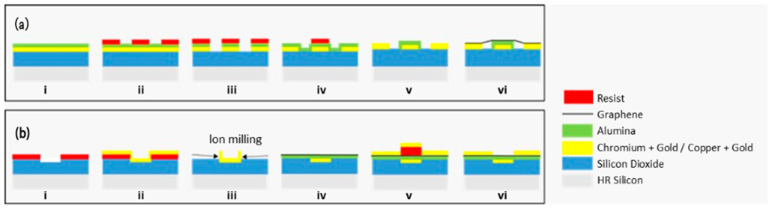
Diagram of the two main processes reported in this work, with and without planarization, (**a**,**b**), respectively. (**a**) (i) high resistivity (HR) silicon wafer with chromium + gold and alumina on top; (ii) e-beam lithography to define source, drain, and gate contacts by ion milling; (iii) device after ion milling; (iv) e-beam lithography to define the gate oxide after the growth of fresh alumina by ALD (after the resist removal by O_2_ plasma, and alumina by wet etch); (v) result after the patterning of the gate oxide by ion milling; (vi) final device with graphene. (**b**) (i) device after e-beam lithography and inductively coupled plasma (ICP) reactive ion etching (RIE); (ii) after the chromium gold deposition; (iii) after the lift-off, showing the ears to be removed by ion milling, to planarize the device; (iv) after the graphene transfer; (v) after the e-beam lithography and copper + gold deposition to define the source/drain electrodes by lift-off; (vi) final device.

The diagram of the two fabrication processes reported in this manuscript can be seen in Figure 1.

### 2.1. Graphene Device Fabrication Process with CPD

**Figure 2:**
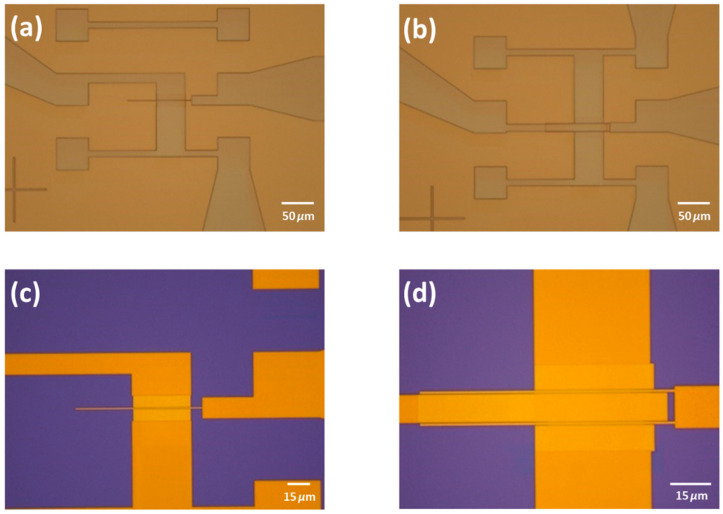
First steps of the fabrication process with (**a**,**b**) showing an optical image of the lithography and (**c**,**d**) showing the optical micrograph after the ion milling and the resist removal by an O_2_ plasma (intermediate step between (iii–iv) in Figure 1).

The fabrication of the bottom-gate devices without planarization began with an HR silicon wafer, used to minimize parasitic capacitances from the substrate, on which 500 nm of SiO_2_ was grown via plasma-enhanced chemical vapor deposition (PECVD). Subsequently, 3 nm of chromium and 97 nm of gold were deposited by sputtering. To define the contacts using ion milling, 10 nm of alumina was sputtered on top of the gold to facilitate the removal of the photoresist. After alumina deposition, the sample was prepared for electron beam (e-beam) lithography. The wafer was coated with AR-N 7520.18, and e-beam lithography was performed to pattern the source, drain, and gate contacts. The contacts were then patterned using ion milling at an angle of 130 degrees, followed by 165 degrees (relative to the surface) to remove redeposited metal “ears”. The photoresist was stripped using an oxygen plasma, and an aluminum etchant (Fujifilm 16:1:1:2 aluminum etch) was employed to remove the alumina, resulting in a clean surface devoid of resist residues. These steps are shown in Figure 2.

**Figure 3:**
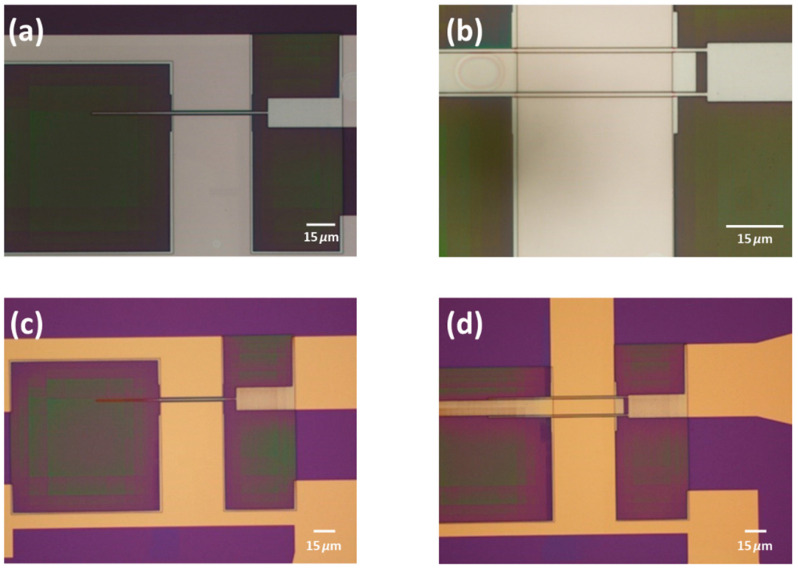
(**a**,**b**) An optical image of the lithography performed to pattern the gate dielectric (alumina), and (**c**,**d**) show the optical image after the ion milling. To note, since the gate is very thin, some e-beam resist was left to act as anchor of the e-beam resist responsible for the patterning of the gate dielectric.

**Figure 4:**
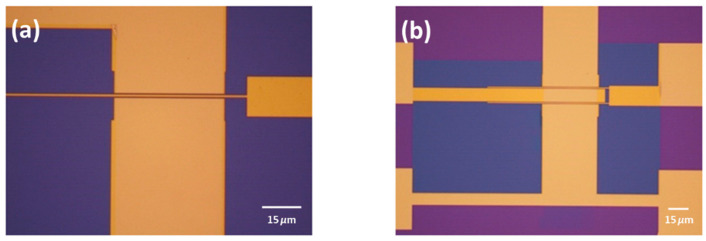
(**a**,**b**) Optical photograph of the device after the resist removal with the oxygen plasma.

Next, 10 nm of Al_2_O_3_ was deposited by ALD to serve as the gate dielectric. The dielectric was patterned using ion milling after an e-beam lithography with AR-N 7520.18. The photoresist was then removed using an oxygen plasma, as illustrated in Figure 3 and Figure 4.

**Figure 5:**
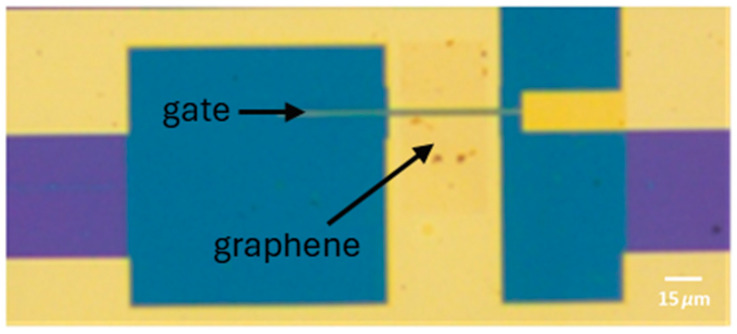
Optical image of the graphene lying in the channel of the device.

CVD graphene grown on copper foil was transferred onto the top of the structures using a PMMA-assisted wet transfer, as reported by our group in [10]. Shortly prior to the transfer, an O_2_ plasma treatment was applied to the back side of the copper foil (which contains the graphene protected with the PMMA) to remove the graphene from that side. The copper foil was then etched in an iron chloride solution (0.5 M). Subsequently, the graphene-PMMA stack was transferred to an HCl (2%) solution to remove iron chloride contaminants and then to water before the final transfer to the substrate. Before this, the substrate surface was dehydrated and primed using a vapor prime oven to promote the adhesion of the graphene to the final substrate. After the transfer, the graphene was dried at room temperature. The PMMA was then removed using acetone. Finally, the graphene was patterned using an O_2_ plasma beam following optical lithography with AZ1505. The resist was removed using acetone overnight. In Figure 5, it is possible to observe the graphene lying in the graphene channel.

**Figure 6:**
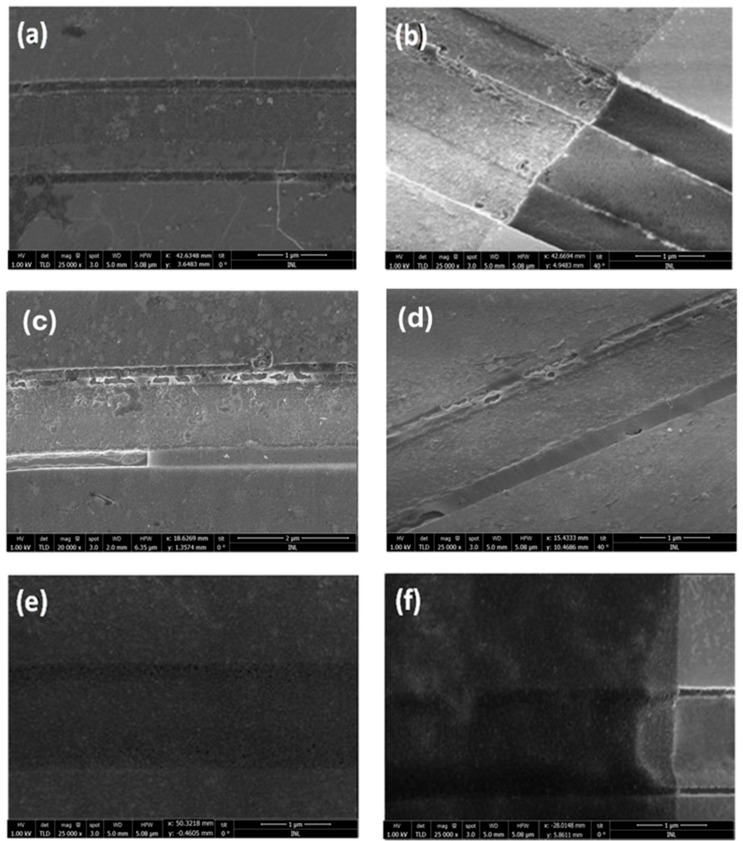
SEM images of graphene after the transfer at 1 kV. (**a**) After the transfer without the patterning, (**b**) after the patterning with graphene following the topography of the contacts, (**c**,**d**) suspended graphene patterned and dried in air, evidencing the cracks, and (**e**,**f**) after the patterning and dried with critical point dryer.

Due to the small gaps between the source and drain contacts (as shown in Figure 6, mandatory for RF devices), the drying process is critically important. Two approaches were tested with the continuous graphene film after patterning (using O_2_ plasma, as it was discovered that graphene only breaks after patterning, i.e., after the removal of the photoresist, not after the removal of the PMMA used for transfer).

In the first approach, the sample was dried in air. In the second approach, critical point drying was used. In the first approach, the surface tension of acetone (during the drying process) resulted in damage to the graphene at some sites, making it unsuitable for RF device fabrication (see Figure 6c,d). For thicknesses above approximately 500 nm, the graphene tends to conform to the surface of the contacts rather than remaining suspended (see Figure 6b), which mitigates the impact of the drying process. This observation supports the use of coplanar structures in sensor fabrication, where graphene on the sidewalls enhances sensor sensitivity [11]. The performance of the fabricated devices is shown in the next section.

In contrast, the second approach, using critical point drying, allowed the graphene to remain suspended due to the uniform drying process (see Figure 6e) and Figure 6f.

Since the use of critical point drying (CPD) poses significant challenges in handling delicate samples, increases the risk of contamination, and as will be shown later, results in devices with limited performance, a new method is proposed. Furthermore, because the graphene must remain immersed in ethanol after the graphene transfer and cannot be allowed to dry at any stage, the transfer and immersion steps become complex and increase the risk of damaging the graphene. To address these issues, a planar bottom-gate topology was developed, offering greater robustness, compatibility with standard lithographic techniques, and improved scalability.

### 2.2. Graphene Device Fabrication Process with Surface Planarization

**Figure 7:**
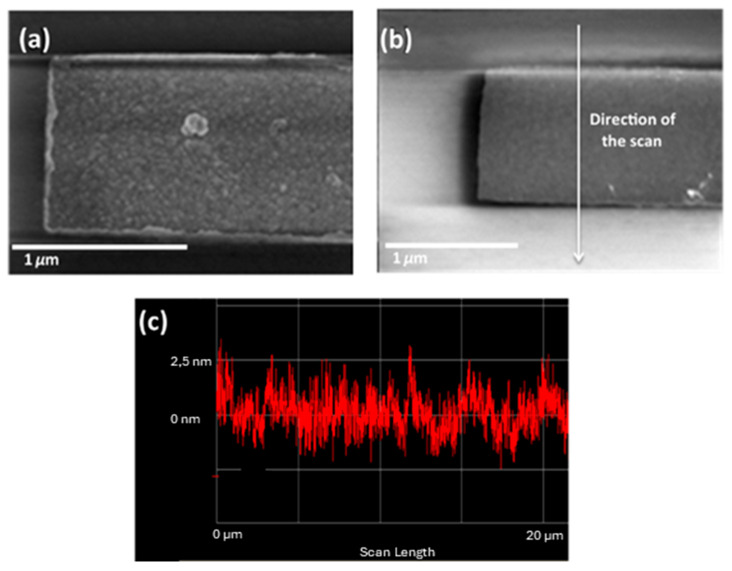
SEM images of the gate structures showing the metal ears (**a**) and after the planarization (**b**). (**c**) Profilometer measurement of the gate after the planarization.

The gate contact was buried within the SiO_2_, and its surface was partially planarized using ion milling at a grazing angle, leading to a reduction in topography by a scale factor of 10. In this way, the graphene is transferred onto a flat surface, avoiding mechanical stress or rupture and thus preserving its characteristic and essential properties—such as high electron mobility—that are crucial for the optimal performance of RF devices. Initially, 500 nm of SiO_2_ was grown on a high-resistivity silicon wafer via PECVD. The gate contact was defined using e-beam lithography, involving the sequential spin coating of AR-P 617.03 and AR-P 6200.09 resists. These resists were selected to simplify the lift-off process for the gate metal. An Inductive Coupled Plasma Reactive Ion Etching (ICP RIE) was performed to open the SiO_2_ trenches without compromising the resists for the lift-off process. Without removing the resist mixture, 3 nm of chromium and 67 nm of gold were sputtered onto the sample. The metal was subsequently lifted off using a solution of *N*-ethylpyrrolidone (NEP) with ultrasonic agitation. Due to the lift-off process, lift-off ears were observed, as shown in Figure 7a. These lift-off ears were removed by ion milling at a 174-degree beam angle (relative to the surface, resulting in a flat surface suitable for graphene transfer (see Figure 7b,c). Note that in (c), it is possible to see only small ears around 4 nm, which could be assumed as a flat surface for graphene.

**Figure 8:**
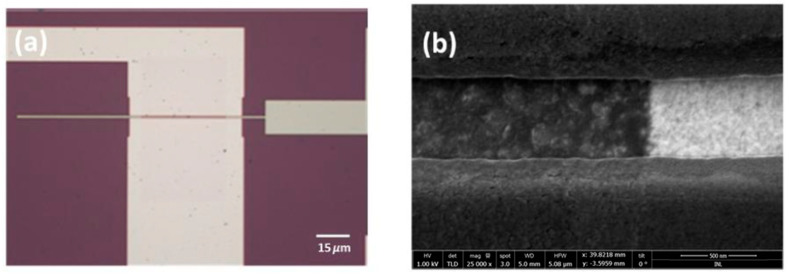
(**a**) Optical image of the final device, and (**b**) SEM image of the final device showing graphene without cracks.

**Figure 9:**
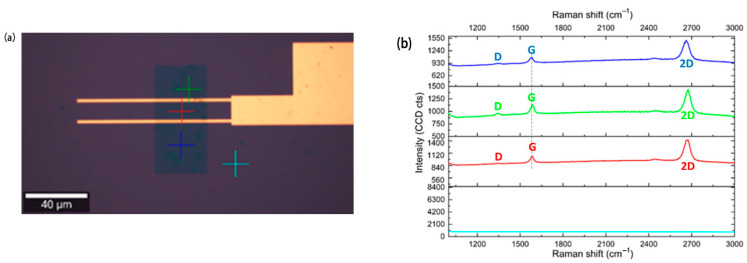
Raman spectra of the monocrystalline graphene used in the fabricated devices, (**a**) Raman spectra sites, and (**b**) Raman spectra acquired with a 532 nm laser (0.5 mW) using a confocal Raman microscope and a 50× objective. D: defect. G: graphene. 2D: 2D peak. It is possible to evaluate the correct patterning since, out of the graphene channel, there is no graphene signature.

After planarization, 10 nm of alumina were deposited using ALD. Standard optical lithography was then employed to selectively remove the alumina via wet etching (Fujifilm 16:1:1:2 aluminum etch) to expose areas for probe contact. Graphene was subsequently transferred onto the surface and patterned with the previously described method. The source/drain electrodes were defined using e-beam lithography with a Poly (methyl methacrylate) (PMMA) bilayer (AR-P 617.03 + AR-P 679.04). Following this, 5 nm of copper and 50 nm of gold were sputtered and then lifted off using acetone. Copper was chosen as the adhesion layer because sputtered chromium, when used with gold, exhibited higher contact resistance compared to copper. The final device can be observed in Figure 8. It is important to note that after the graphene transfer in both fabrication processes, the quality of the graphene was assessed via Raman spectroscopy, as shown in Figure 9.

## 3. Performance Assessment of the Proposed Wafer-Level Fabrication

The proposed fabrication methods were employed to produce sets of GFETs. The performance assessment of these transistors is reported in this section of the manuscript. Graphene purity assessment is performed by resorting to Raman spectroscopy. Then, the electrical performance of the transistors is verified for low- and high-frequency signals.

### 3.1. Graphene Quality Assessment

In the previously reported fabrication processes, monocrystalline graphene was used to achieve a high yield in the fabricated devices. The graphene was carefully transferred onto the substrates to maintain its crystalline integrity. To ensure the quality and continuity of the graphene layer, Raman spectroscopy was employed. The Raman spectra of the graphene layer can be observed in Figure 9.

As observed in the Raman spectra, a blue shift occurs, specifically with the G peak shifting to a higher wavenumber. This shift indicates the presence of p-type doping [12]. The p-doping may originate from the fabrication process (PMMA contaminants or photoresist [10]) or from contaminants in the ambient environment that interact with the graphene surface [13]. Furthermore, the Raman spectra confirm that the graphene is predominantly monolayer (evidenced by the I_2D_/I_G_ > 2), although defects are present, as evidenced by the appearance of the D peak. The successful patterning of the graphene is demonstrated by the absence of graphene outside the designated channel region, as seen both in the corresponding images and Raman spectra. The graphene mobility was extracted through the transfer curve fitting method, being 900 cm^2^ V^−1^ s^−1^ for holes and 1100 cm^2^ V^−1^ s^−1^ for electrons [14].

### 3.2. Low-Frequency Electrical Performance

#### 3.2.1. Graphene Device Fabrication Process Without Surface Planarization (CPD)

**Figure 10:**
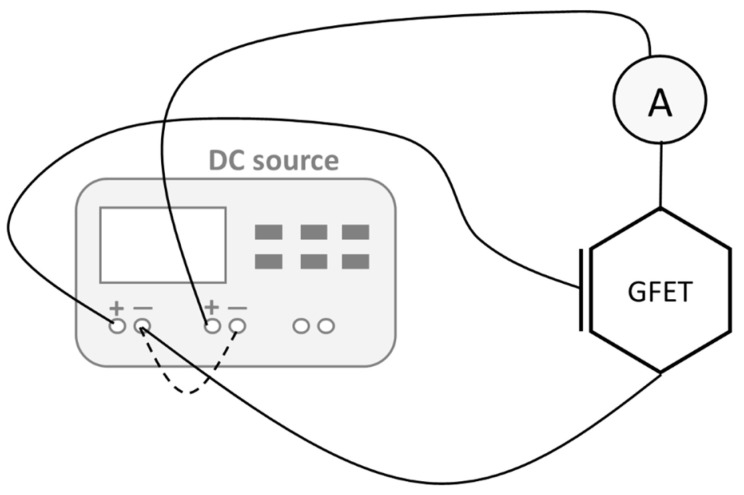
Measurement setup to extract the transfer curve of GFET transistors.

**Figure 11:**
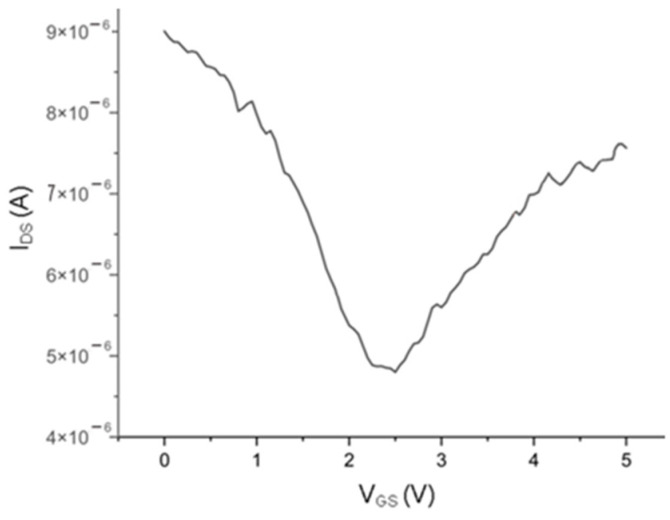
I_DS_ vs. V_GS_ and $g_{m}$ characteristic curves of a device with W = 34 µm and L = 1.100 µm (0.95 µm of gate channel and 0.075 µm of drain/gate and source/gate overlap) measured at a V_DS_ of 100 mV.

The DC measurements were performed to obtain the I_DS_ and the respective $g_{m}$. To do this, two DC sources were required, one connected to the source and drain contacts and another to the gate and source contacts, as shown in Figure 10. The measurements were performed by measuring the I_DS,_ changing one of the DC sources, and keeping the other constant. The results for a device with W = 34 µm and L = 1.100 µm are shown in Figure 11. The maximum extracted $g_{m}$ is 4 µS.

#### 3.2.2. Planar Buried Bottom-Gate Topology Improved

**Figure 12:**
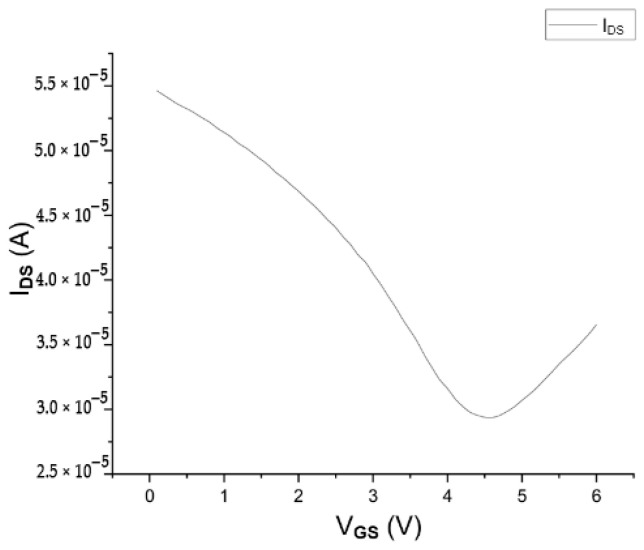
Transfer curve of a device with L = 1.102 μm and W = 33.82 μm. V_GS_ vs. I_DS_ curve for a V_DS_ of 10 mV.

The low-frequency electrical properties of GFETs fabricated with the planar buried bottom-gate topology were evaluated with the same procedure as the previously reported CPD-fabricated GFTETs. The data derived from these measurements (transfer curves) are illustrated in the following figure for a device with L = 1.101 μm and W = 33.82 μm, similar dimensions to the previously shown device in Figure 12.

The Dirac voltage of the device is observed at approximately 4.5 V (positive voltage), indicating p-type doping, as previously suggested during the Raman evaluation. A g_m_ of 10 µS was obtained, which is bigger than the one achieved in 3.2.1, showing an increase in the device performance for DC applications. To note that due to the different graphene sample used to fabricate the devices, the p-doping is more prominent in the planar buried bottom-gate topology process, as shown in the shift of the Dirac point in Figure 12 when compared with Figure 11.

### 3.3. High-Frequency Electrical Performance

**Figure 13:**
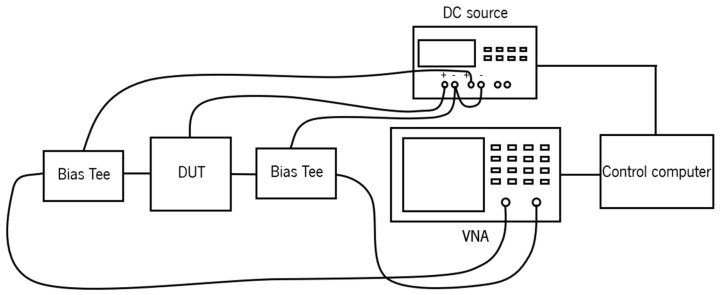
Measurement setup used to extract the FOMs of the GFETs.

The electrical performance is evaluated through the extraction of the figure of merit (FOM) for graphene field-effect transistors (GFETs). This setup incorporates two bias tees to separate direct current (DC) and radio frequency (RF) signals, enabling the simultaneous application of a DC bias voltage and an RF signal to the device. A DC power supply is employed to provide the necessary gate and drain bias voltages, thereby biasing the GFET into the desired operational region. A E5071C VNA (Keysight, Santa Rosa, CA, USA) is used to generate and measure high-frequency RF signals, allowing for the determination of the device’s scattering parameters (S-parameters), which are critical for the calculation of the transistor’s FOM. Additionally, a control computer interface is employed to regulate the gate’s DC voltage and to facilitate the extraction and analysis of the S-parameters. The measurement setup is represented in Figure 13.

#### 3.3.1. Graphene Device Fabrication Process with CPD

**Figure 14:**
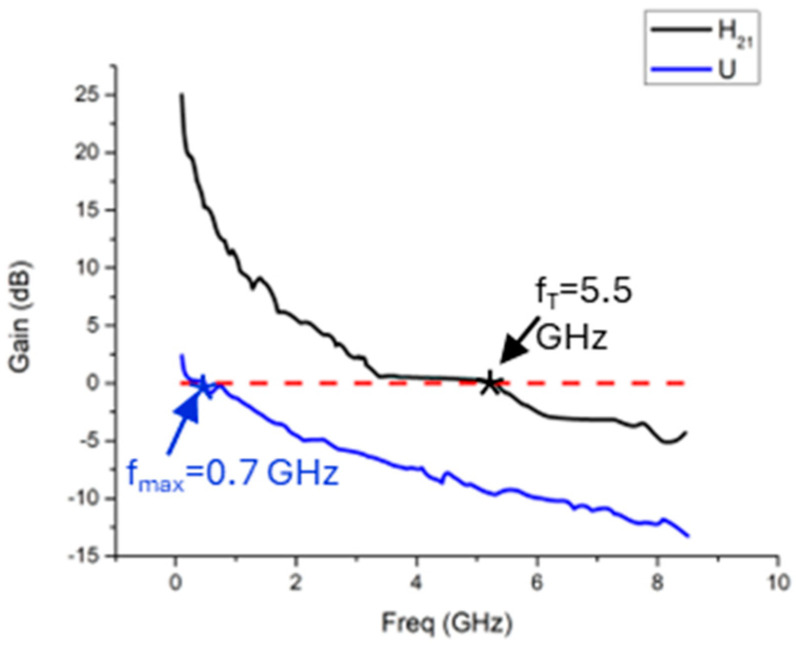
Gain (dB) vs. Freq (GHz) of a device with W = 34 µm and L = 1.100 µm and a La (access length) of 85 nm and the extracted fT and fmax.

To evaluate the performance of the devices fabricated using the CPD method, extrinsic RF characterization was performed. Figure 14 illustrates measured S-parameters (a) and the extracted FOMs (b). In this device, an extrinsic cut-off frequency (fT) of 5.5 GHz and a maximum oscillation frequency (fmax) of 0.7 GHz, measured at a VDS of 3 V and a VGS of 5 V, were demonstrated. These values were obtained without employing a global back gate, suggesting a high level of p-doping in the graphene membrane. This fabrication method achieved a high yield of operational devices, with approximately 70% exhibiting RF functionality.

#### 3.3.2. Planar Buried Bottom-Gate Topology

**Figure 15:**
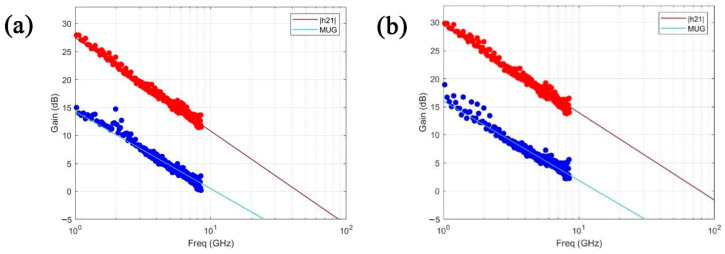
RF performance of the de-embedded devices. H21 and MUG of a device with an L = 1.101 µm (**a**,**b**) of a device with a L = 1.136 µm and a La of 54.35 nm. The f_max_ and f_T_ of (**a**) are 11 GHz and 44 GHz, respectively, and in (**b**) are 14 GHz and 80 GHz, respectively.

Considering the DC performance of the devices, the RF performance was also evaluated. Notably, de-embedded structures were fabricated to extract both the intrinsic fT and extrinsic fmax, isolating the contributions from parasitic elements external to the device that significantly impact its performance. The open structure consisted of a device similar to the fabricated transistors but without graphene in the channel. The short structure, in contrast, was fabricated with all electrodes connected. Figure 15 shows the RF performance of two distinct devices after the de-embedding, showing the extrinsic and intrinsic performance of the transistors. As concluded earlier, and as expected, the device with a small La exhibited a better performance than the one without La.

## 4. Performance Assessment of Fabricated RF Building Blocks

To assess the viability of the proposed methodology, both active and passive devices were fabricated and integrated into more complex circuits, demonstrating the potential of this process for use in systems capable of performing advanced functions.

### 4.1. Performance Assessment of Fabricated Active Devices

The fabricated devices underwent validation through integration into various circuits built with active devices to demonstrate their suitability for real-world applications. The circuits were selected to cover a representative range of functionalities, from basic logic operations to more complex nonlinear applications. For instance, standard inverters were implemented to evaluate the devices’ switching capabilities and overall performance in digital applications. Additionally, the devices were tested in nonlinear circuits, such as mixers and frequency doublers, which are critical in RF and communication systems. Mixers were employed to analyze the devices’ behavior in frequency conversion, and frequency doublers were tested to provide insights into harmonic generation and efficiency under nonlinear conditions. These tests helped characterize the devices under different operating conditions, providing a comprehensive understanding of their potential for practical use.

#### 4.1.1. Transistor Integrated to Perform Digital Operations

**Figure 16:**
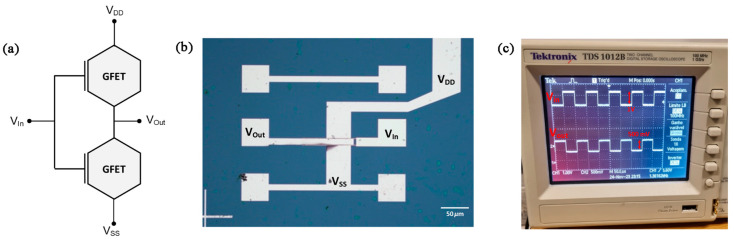
(**a**) Inverter schematic with GFET. (**b**) Equivalence of the fabricated device with the schematic shown in (**a**). (**c**) Measurement of a graphene inverter with a V_DD_ of 8V showing a V_out_ of 500 mV of amplitude swing (channel 2) for a V_in_ of 1 V swing centered in 5.7 V.

The graphene inverters, which serve as the fundamental building blocks for RF graphene oscillators, were fabricated and validated to evaluate their RF performance and suitability for integration. To test the inverter, a square wave with a 1 V amplitude swing centered at 5.7 V (chosen because of the Dirac voltages since V_Dirac_ are far from 0 V) was applied to the inverter’s input (V_in_). The schematic of the inverter is shown in Figure 16a, while the fabricated device is depicted in Figure 16b. The inverter was powered by a supply voltage (V_DD_) of 8 V. For the applied V_in_, the measured output voltage (V_out_) was 500 mV, corresponding to an inverter gain (A_v_) of 0.5. The output signal is centered at 0 V due to the use of a bias tee, which isolates and recovers the AC component of the output. Figure 16c presents the oscilloscope capture, displaying both the input and output signals, further confirming the inverter’s operation and performance.

Despite the favorable f_T_ and f_max_ reported earlier, the measured inverter did not exhibit a gain greater than 1 (A_v_ = 0.5), a criterion essential for oscillation according to Barkhausen’s stability criterion. This limitation may arise from the selected device or the design of the inverter itself. Efforts were made to measure the fabricated ring oscillator to validate its functionality; however, no output was observed when using the RF probes. To ensure proper operation of the ring oscillators, it is necessary to increase the gain of the inverter. However, due to the high p-doping observed in the fabricated transistors, increasing the supply voltage (V_DD_) to boost the gain is not a viable solution, given the breakdown field of the materials. In fact, the scaling of electronic circuits typically involves a reduction in the supply voltage to ensure that the electric field in scaled devices remains below the breakdown field of the material [15]. In the following subsection, some nonlinear blocks, such as a frequency doubler and a mixer, will be analyzed.

#### 4.1.2. Transistor Integrated to Perform Analog Operations

Based on the RF performance of the transistors presented in the previous sections, both an RF mixer and an RF frequency doubler were implemented to further validate their suitability for GHz-range signal processing. To demonstrate the mixer functionality, the device was biased with a drain-source voltage (V_DS_) of 4 V. Two RF signals, 3.2 GHz and 3.0 GHz, each with a power level of 0 dBm, were combined and applied to the gate of the transistor using a bias-tee, which enabled the superposition of RF and DC signals while preventing direct interaction between the DC and RF sources. A DC gate voltage of 2.5 V was applied, corresponding to the Dirac point of the device.

The output spectrum exhibited the expected frequency components resulting from the mixing of the two input signals. Specifically, signals were observed at 6.0 GHz (−76.3 dBm), 6.2 GHz (−67.2 dBm), and 6.4 GHz (−68.35 dBm), confirming the feasibility of using the proposed device architecture as an RF mixer. The RF power levels were carefully selected to ensure device integrity, considering the sensitivity of the graphene-based active layer.

The frequency doubler was characterized using a similar experimental setup, with the primary distinction being the use of a single RF source and the absence of a signal combiner. The input signal was directly injected into the gate via an RF probe, while the output was collected through a second probe. The device was again biased at V_DS_ = 4 V with a gate voltage of 2.5 V. An input signal of 3 GHz at 0 dBm produced an output component at 6 GHz with a measured power of −72.24 dBm, demonstrating the device’s capability to operate as an RF frequency doubler.

### 4.2. Performance Assessment of Fabricated Passive Devices

RF systems are comprised of both active and passive components. After testing and validating active components in the previous sections, the focus now shifts towards passives and the study of the viability of graphene as an enabler for passive RF devices. In recent years, biomedical engineering has experienced remarkable advancements, which were fueled by the integration of emerging materials and wireless technologies. One of those materials is graphene, where, in biosensing, for example, using functionalized graphene has allowed for the development of ultrasensitive biosensors [16,17,18]. These advancements can revolutionize the field through highly sensitive, real-time detection of biological analytes.

An application of functionalized graphene can be its implementation within antennas. Antennas are necessary for wireless communication, information exchange, and even wireless power transfer. Through the integration of functionalized graphene as the biosensing element in antennas, it should be possible to obtain a wireless biosensor. When functionalized graphene is exposed to a target analyte, that interaction leads to changes in its electrical properties. These changes, in turn, modulate the signal transmitted by the antenna, allowing for the detection and quantification of the target analyte. Through this technique, it should also be possible to combine the benefits of both and obtain a wireless backscatter biosensor. The development of such a system should allow to enable wireless, remote, and non-invasive biosensing with high sensitivity and accuracy.

In this section, the development of a graphene backscatter antenna will be discussed. The design and fabrication of the device will be discussed, as well as the underlying principles and shedding light on the future prospects of such a device.

#### 4.2.1. Wireless Sensing Readout Working Principle

Since graphene is a material with tunable electrical properties that can be controlled by adjusting its chemical potential, it is possible to tune its properties as previously demonstrated and characterized. The interaction of graphene with its surrounding environment, such as target analytes in the case of biosensors, causes its chemical potential to change, thus also changing its impedance. This means that changing the chemical potential of graphene provides valuable information about the interaction between the environmental properties and the graphene surface. By monitoring the impedance change in the graphene biosensor, it is possible to detect and quantify these changes, as is the case with analytes in biosensors.

The tuning of graphene’s chemical potential can be achieved through several methods. In the example of biosensors, the most relevant ones are the target analytes coupling with graphene and the voltage gating. Therefore, the change in impedance of graphene biosensors can be effectively modeled or translated by a corresponding change in the gate voltage applied to the graphene layer. These changes in impedance can later be correlated so that a relationship between impedance and gate voltage through experimental calibration can be established, allowing us to interpret the electrical responses of biosensors.

Using RF for wireless power transfer with the objective of powering a biomedical device or sensor and receiving a readout has been the focus of various research works [19,20,21,22]. An example is shown in [20], where a solution of wireless power with tracking is presented. It should be possible to implement a biosensor readout by modulating the feedback signal.

In this section, an antenna is implemented with a load that is graphene. To mimic the graphene biosensing capability, graphene will be tuned through voltage gating. Since gating graphene changes its impedance, it should be possible to modify and control the reflected power. Therefore, since graphene is directly connected to the antenna, the amount of reflected power should change according to graphene’s impedance. For this experience, the device was fabricated with the surface planarization technique with ion milling.

#### 4.2.2. Sensing Antenna Design

**Figure 17:**
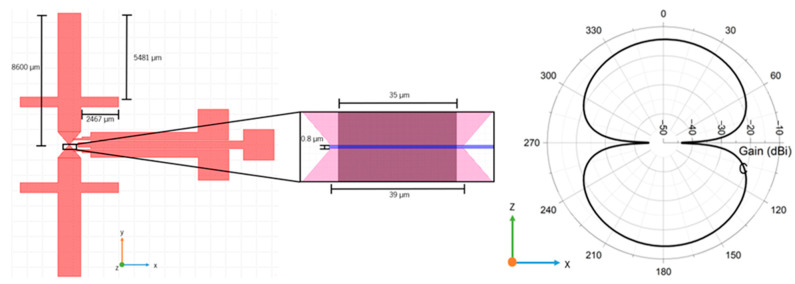
On the left is shown the dipole antenna designed with graphene to obtain a backscatter readout; the inset shows the area with the graphene, as well as its gate and dimensions. On the right is shown the simulated radiation diagram of the antenna.

The antenna is similar to a dipole, where two stub-like structures are added on each dipole arm to increase its frequency range. To limit the total size of the antenna, the chosen minimum operation frequency was 3 GHz. To control the resistance of graphene through gating, at least two DC contacts are necessary: a reference and a gate. The antenna is shown on the left side of Figure 17, as well as its dimensions.

This antenna was simulated, allowing us to determine its operation frequency at 3 GHz. On the right side of Figure 17, the cross-section of the E-plane radiation diagram at this frequency is shown, where a maximum gain of −14.35 dBi (−11.827 dBi at 6 GHz) can be observed, while its directivity is approximately 3.5 dBi across the simulation frequency range.

#### 4.2.3. Backscattering Antenna Readout

**Figure 18:**
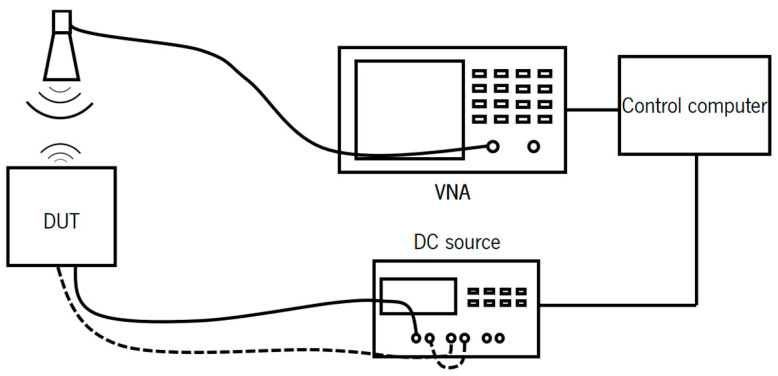
Diagram of the measurement setup used for the measurement of the backscatter of the antenna with graphene on the bottom.

To measure this variation in reflected power, the VNA was calibrated with the Short-Open-Load-Through (SOLT) calibration. To more easily measure the variation in the reflection of the DUT, the load standard was replaced by the antenna. The objective of this modification was to set the reference (the zero reflection) to the background environment. In this way, any change in the graphene load placed at the antenna port will be less difficult to detect. The measurement antenna used was the PE9887-11 horn antenna (Pasternack, Irvine, CA, USA), which has an operating frequency from 4.9 GHz to 7 GHz. The fabricated sample was placed on a wooden table, the horn antenna was placed on top of it, and the DC pads were contacted with needles. The measurement setup block diagram is shown in Figure 18.

**Figure 19:**
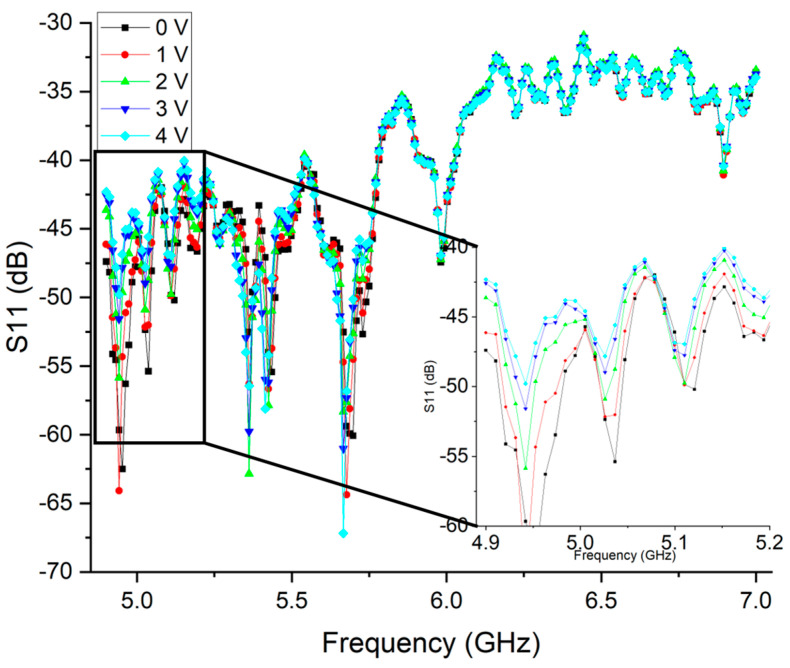
Measured S_11_ in dB of the backscatter by changing the graphene voltage from 0 to 4 volts in the working range of the measurement antenna.

The measurement was performed by changing the gate voltage and measuring the S_11_ through the VNA. The applied voltage ranged from 0 V to 4 V, and the measurement was performed in 1 V steps. The measured values are shown in Figure 19.

These results show the measured S_11_ for different applied voltages. The inset shown allows us to observe that there is a change in the S_11_ for different applied voltages. At around 4.95 GHz, it is possible to observe that the S_11_ changes from around −65 dB (red curve) up to around −50 dB (light blue curve). The same behavior can be observed for the remaining frequency points, although with a lower variation. These results show that it is possible to control the backscatter of the device by applying a gating voltage to graphene.

## 5. Conclusions

This work demonstrated the successful development and implementation of two wafer-scale fabrication processes tailored for graphene-based RF devices. The fabricated GFETs exhibited promising RF performance, with intrinsic f_T_ reaching 14 GHz and fmax up to 80 GHz. The compatibility of the proposed processes with CVD-grown graphene reinforces the scalability of the technology and opens the possibility of extending the methodology to other 2D materials, such as MoSe_2_. The flexibility of the approach allows for the development of hybrid systems and heterogeneous integration, particularly in applications requiring conformal or biocompatible electronics.

The process flow, device topologies, and layout designs developed in this work provide a viable path forward for integrating graphene RF devices with sensing and actuation functionalities. In particular, the fact that graphene remains exposed throughout much of the fabrication process enables direct functionalization, making the proposed platform highly attractive for biosensing and bio-interfacing applications. An antenna with graphene was developed, enabling passive wireless readout. Through this work, it was demonstrated that the measured S_11_ changed according to an applied gate voltage, allowing it to mimic a graphene sensor. The backscatter readout achieved a maximum variation of around 15 dB.

This study represents a significant step toward enabling reliable, reproducible, and scalable integration of graphene into RF systems. It contributes to the broader effort of transitioning 2D materials from laboratory-scale demonstrations to real-world technologies with impact across communications, sensing, and biomedical domains.

## Data Availability

The original contributions presented in this study are included in the article. Further inquiries can be directed to the corresponding author.
